# Attenuation of Autoimmune Phenomena in a Patient with Autoimmune Polyglandular Syndrome Type 1

**DOI:** 10.1155/2021/6009141

**Published:** 2021-12-13

**Authors:** Jill D. Jacobson, Julia R. Broussard, Courtney Marsh, Brandon Newell

**Affiliations:** ^1^Division of Endocrinology and Diabetes, Department of Pediatrics, Children's Mercy, University of Missouri-Kansas City School of Medicine, Kansas, MO 64111, USA; ^2^Division of Reproductive Endocrinology and Infertility, Department of Obstetrics and Gynecology, University of Kansas School of Medicine, Kansas, KS 66160, USA; ^3^Division of Dermatology, Department of Pediatrics, Children's Mercy Hospital, University of Missouri-Kansas City School of Medicine, Kansas, MO 64111, USA

## Abstract

Autoimmune polyglandular syndrome type 1 (APS1) is a progressive life-threatening illness with no known cure. Current treatments involve replacement of the hormone deficiencies that result from autoimmune destruction of multiple endocrine organs. We report on a girl whose disease was progressing rapidly until she began on immunosuppressive agents. A healthy 6-year-old girl with no remarkable medical history presented with new onset hypocalcemic seizures and primary hypoparathyroidism. Howell-Jolly bodies consistent with autoimmune hyposplenism were also noted. Genetic testing revealed compound heterozygosity for 2 disease-associated variants in the autoimmune regulator (AIRE) gene. She later developed elevated liver enzymes, primary adrenal insufficiency, and alopecia totalis. Serologic testing revealed antibodies to 21-hydroxylase, intrinsic factor, and smooth muscle. Hydrocortisone was initiated for adrenal insufficiency. Shortly afterwards, her liver enzymes normalized, and her smooth muscle antibody levels began to decline. Serologic testing performed at age 11 revealed seropositivity for glutamic acid decarboxylase (GAD) antibodies, antinuclear antibodies, and Sjögren syndrome A (SSA) antibodies. At age 12, she was given 2 doses of rituximab. Hair loss rapidly progressed to alopecia totalis and then to alopecia universalis, at which time oral methotrexate treatment was initiated. For the past 7 years while on glucocorticoid and methotrexate treatment, our patient has displayed normalization of 2 antibodies, a lack of progression to additional autoimmune diseases, and experienced reversal of alopecia universalis.

## 1. Introduction

Autoimmune polyglandular syndrome type 1 (APS1), also known as APECED (autoimmune polyendocrinopathy candidiasis ectodermal dysplasia), is a rare but serious disorder associated with progressive autoimmune destruction of multiple endocrine and nonendocrine organs. APS1 has a high mortality rate. The median age at death ranges from 5 to 34 years, with deaths attributable to endocrinopathies, malignancy, acute hepatitis, and infection [1]. APS1 is caused by biallelic variations of the gene encoding the autoimmune regulator (AIRE), resulting in dysfunction of regulatory T cells, impaired immunologic tolerance to self-antigens with subsequent development of multiple autoimmune conditions [2]. Immunosuppressive therapies have been generally reserved for life-threatening features of APS1. To the best of our knowledge, this is the first case report demonstrating normalization of serology, reversal of an autoimmune condition, and prolonged lack of progression of autoimmune damage in APS1.

## 2. Case Report

The patient was a previously healthy 6-year-old girl of northern European descent whose only concerns had been enlarged tonsils, chronic constipation, and slow growth. She had no other health problems. There was no family history of endocrine or immunologic diseases. While watching television, she developed a grand mal seizure and became apneic. She was emergently transported to Children's Mercy Hospital. Initial physical examination was unremarkable except for short stature, with weight 20 kg (37.7 percentile) and height 104.6 cm (less than the 1^st^ percentile). Family history revealed that the patient's mother is 162.6 cm tall and father is 177.8 cm tall; midparental height is 167.7 cm (50^th^ percentile).

She was found to have a critically low total calcium of 1.1 mmol/L (normal range 2.2–2.5 mmol/L) and a blood glucose of 3.6 mmol/L (normal range 3.6–6.1 mmol/L). Her phosphorus was elevated at 3.6 mmol/L (1–1.9 mmol/L), and magnesium was low at 0.49 mmol/L (0.66–0.94 mmol/L). Initial iPTH level was low at 7 ng/L (10–89 ng/L), and subsequent iPTH levels remained low. She was diagnosed with primary hypoparathyroidism. She received intravenous calcium chloride and magnesium sulfate. Computed tomography of the head was normal. An extensive endocrine workup revealed that she had Howell-Jolly bodies consistent with autoimmune hyposplenism, a condition frequently seen in APS1 [3]. No other autoimmune deficiencies were noted at that time. Karyotype was 46, XX. Evaluation of 22 q 11 variants was normal. A growth hormone (GH) stimulation test was performed during initial admission. Her peak GH level was 12.8 ng/mL (normal >10 ng/mL).

With the documentation of two unusual autoimmune findings, genetic testing for AIRE gene was performed. The patient was found to be a compound heterozygote for 2 known disease-causing variants. The first was a nucleotide change of C > T in exon 6 of the AIRE gene resulting in the substitution of the normal arginine codon with a stop codon at position 257. This mutation is denoted R257X or Arg257Term. The second mutation was a 13 base-pair deletion in exon 8, beginning in codon leucine 323 and resulting in a change from leucine to serine, followed by a frameshift and premature stop codon 50 residues downstream (denoted c.967 979del13 and p.Leu323SerfsX50). Thus, she was heterozygous for R257X and c.967 979del13. Both are common, independently recurring mutations in APS1 [4]. The 13-base deletion has been published with various nomenclature (c.965 977del13 or p.Cys322fsX5l).

**Figure 1:**
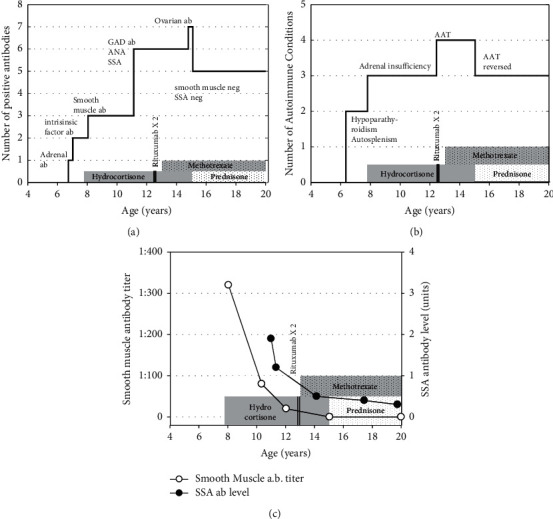
Timeline of development of autoantibodies (a), autoimmune processes (b), and smooth muscle and SSA antibodies (c) with time course of known immunosuppressive therapy also shown.

After testing positive for AIRE gene variants, additional serologic testing revealed seropositivity for 21-hydroxylase antibodies (a marker for adrenal autoimmunity) and positivity for intrinsic factor autoantibodies (a marker for atrophic gastritis).  Figure 1(a) shows her positive serology over time, along with her immunosuppressive medications. Antibody testing for thyroid disease, type 1 diabetes mellitus, and celiac disease yielded negative results at that time.

At the age of 6 and a half years, a low-dose ACTH stimulation test showed a borderline peak cortisol of 433 nmol/L (normal >500 nmol/L). ACTH stimulation testing was repeated a year later, at which time, she demonstrated a peak cortisol level of 334 nmol/L, in addition to an elevated renin level. Subsequently, hydrocortisone and fludrocortisone replacement therapy were initiated.  Figure 1(b) shows the number of autoimmune conditions over time along with her immunosuppressive medications.

Because of persistent short stature, she underwent repeat GH stimulation testing around age 9. Her peak GH level was 11.8 ng/mL, demonstrating GH sufficiency again. However, as her height was below the 3^rd^ percentile, growth hormone therapy was initiated with excellent response. She ultimately achieved an adult height of 161.5 cm, within the range of her midparental height.

Her liver enzymes were modestly elevated at this time. Serologic testing revealed positive smooth muscle antibodies, which are associated with autoimmune hepatitis. At age 10, she developed hypertension and nephrocalcinosis and was placed on thiazide diuretics. At the age of 11 years, serologic testing revealed positive glutamic acid dehydrogenase (GAD), antinuclear antibodies (ANA), and Sjögren syndrome antibodies (SSA). Her thyroid antibodies have remained negative throughout her course. Over time, her smooth muscle antibodies and SSA antibody levels have gradually normalized (Figure 1(c)).

At age 12 years, she began to develop patches of alopecia on her scalp, which was distressing to her. We referred her to rheumatology for aggressive management of her APS1. She was begun on rituximab, monoclonal antibody therapy directed at CD20, a B cell epitope. Unfortunately, soon after she received 2 doses of rituximab, her hair loss progressed rapidly to alopecia totalis and then progressed to alopecia universalis.

**Figure 2:**
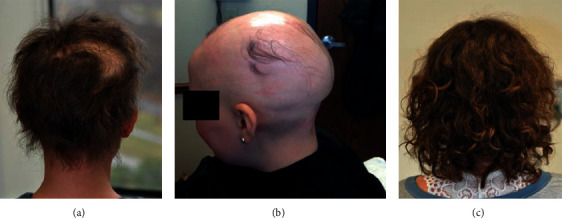
Progression of alopecia: early stages of disease (a), progression of disease (b), improvement in alopecia (c).

The patient was referred to pediatric dermatology, who began with intralesional triamcinolone injections but soon added oral methotrexate. She was begun on 20 mg weekly, but this was increased to 25 mg weekly at age 16, which she continues to date. She was empirically placed on 1 mg folic acid daily and vitamin B12 with the methotrexate therapy. Complete hair regrowth was achieved within a year.  Figure 2 denotes progressive hair loss (2(a) and 2(b)) followed by hair regrowth (2(c)). The patient has tolerated the methotrexate well.

At the age of 15, she was referred to gynecology for fertility discussion. Periods had been regular throughout, and gonadotropins were normal. Ovarian antibodies, known to be nonspecific, were measured at that time and were positive. Midcycle LH was 25 IU/L, and FSH was 7.1 IU/L. Anti-Müllerian hormone (AMH) level was 21.3 pmol/L (normal range for AMH 7.5–91.8 pmol/L). Six months later, the AMH level was noted to be 6.9 pmol/L, suggestive of low ovarian reserve [5, 6]. She then underwent fertility preservation. The procedure was highly successful, with 21 eggs harvested and stored. Surprisingly, five months after the retrieval, AMH levels were found to have normalized at 152.4 pmol/L. Her LH and FSH normalized. Her most recent LH is 0.5 IU/L, and her LH is 1.9 IUL. Cycles have remained regular throughout.

At age 18, a bone mineral density study noted focal areas of severely decreased bone mineral density in the distal femurs. Plain films showed ill-defined lucencies with adjacent sclerosis in the distal femurs, which we attribute to metaphyseal dysplasia, a rare bone condition previously described in 2003 in 2 unrelated patients with APS1 [7].

Six months later, she suddenly developed profound hypokalemia, which was thought to relate to apparent mineralocorticoid excess that has been described in APS1 [2]. This was managed with spironolactone and a reduction in fludrocortisone. A few weeks later, she experienced sudden mental status changes. She was seen emergently and was thought to be in septic shock. Testing for active COVID-infection was negative, but she had IgG antibodies to COVID-and met the diagnostic criteria for multisystem inflammatory syndrome in children (MIS-C). She required fluids and intensive care support. She recovered uneventfully from MIS-C, although she remains with elevated brain natriuretic peptide.

She is currently doing well as a college student at a major university and undergoes frequent laboratory monitoring.

## 3. Discussion

Herein, we describe a patient with autoimmune polyglandular syndrome type 1 (APS1) also known as autoimmune polyendocrinopathy candidiasis ectodermal dysplasia (APECED) with molecularly confirmed disease-causing variants in the AIRE gene. She was diagnosed at a young age in the absence of candida infections because of hypoparathyroidism in the presence of Howell-Jolly bodies, suggestive of autosplenism APS1 [3].

Our patient's clinical course is to the best of our knowledge unique. Early in her course, she was developing seropositivity to various antigens autoimmune conditions at a consistent rate of nearly one per year (Figure 1(a)). She was developing autoimmune conditions at nearly the same rate (Figure 1(b)). In the past 7 years, while receiving physiologic glucocorticoids and methotrexate therapy, she has not developed any new autoimmune problems. Moreover, her alopecia universalis has gone into remission, and her smooth muscle and SSA antibodies have normalized (Figure 1(c)). We are not aware of any reports in the literature of such attenuation of APS1.

Current management of APS1 does not generally involve immunosuppressive therapy. The illness tends to fall in the realm of endocrinologists rather than immunologists. Generally, treatment is directed toward replacing the hormones that are deficient as a direct result of the autoimmune processes. Adrenal disease is treated with glucocorticoid and mineralocorticoid replacement. Hypoparathyroidism is treated with oral calcium supplements and activated forms of vitamin D.

A mouse model of APS1 has been developed [8]. Rituximab and anti-CD4 antibody therapy improve APS1 in the mouse model [8], but these therapies have not been translated to humans. We are aware of no active clinical therapeutic trials.

When immunosuppressive therapies have been used, it is often in the setting of life-threatening features of APS1. A recent article outlined the immunosuppressive agents that have been tried in APS1 patients with severe conditions such as interstitial lung disease, autoimmune hepatitis, tubulointerstitial nephritis, pure red cell aplasia, and severe malabsorption [9]. That series included a summary of 13 previously reported patients, and therapies included high-dose glucocorticoids, cyclosporine A, mycophenolate mofetil, rituximab, monoclonal antibodies directed against CD52, macrolide compounds, and methotrexate [1, 9]. Most patients received multidrug therapy. Results were mixed. Only 1 patient, a patient with malabsorption, was treated with methotrexate. Therapy was combined with high-dose steroids, and remission in malabsorption was achieved [10].

A handful of other reports exist in which immunosuppressive agents have been used in APS1. Successful treatment of ocular keratitis with topical cyclosporine has been reported in two patients with APS1 [11]. In another study, 2 of 3 children with autoimmune hepatitis responded well to glucocorticoids and azathioprine [12]. A young boy with APS1 underwent a liver transplant and had a reduction in autoantibodies when he received a regimen of tacrolimus, prednisone, and mycophenolate mofetil [13].

Patients with isolated autoimmune diseases in the absence of APS1 may experience remissions. Isolated alopecia totalis can have a variable, relapsing, or remitting course. The possibility of spontaneous remission exists with this isolated condition for many years after onset. A similar situation exists with ovarian failure. A recent meta-analysis evaluating pregnancies in patients with premature ovarian insufficiency showed a spontaneous pregnancy rate ranging from 2.2 to 14.2% [14].

This study is limited by the difficulty in identifying which immunosuppressive agent may have offered our patient benefit. The two antibodies that normalized began to decline after the initiation of hydrocortisone, a known immunosuppressive agent. Fludrocortisone was initiated simultaneously. Although fludrocortisone has significant glucocorticoid effects, those effects are thought to be minimal at physiologic doses. After the 2 doses of rituximab at age 12, her hair loss acutely accelerated, resulting in a decision to try other forms of immunotherapy. Although hair loss worsened in close temporal proximity to the rituximab injections, it is impossible to say whether the rituximab exacerbated her symptoms or contributed to her later improvement. Based on the timing of the normalization of 2 antibodies prior to beginning on rituximab and methotrexate, it is possible that administration of glucocorticoids even at physiologic doses may have exerted immune benefits. This case may argue for early and repeated testing for adrenal insufficiency in APS1.

She was switched from a short-acting to a long-acting glucocorticoid after she achieved her adult height. We theorized that a long-acting steroid may suppress the immune system more consistently than short-acting ones. She also received small doses of a plant-derived progesterone, which is compounded and administered in troche form in order to titrate the dose. Several studies show that progesterone has glucocorticoid properties [15–17]. To provide steady release of an additional potentially immunosuppressive agent, we recommended low-dose etonogestrel as an implant.

## 4. Conclusions

We describe a patient with molecularly confirmed APS1 with marked attenuation of her seropositivity and autoimmune phenomena and remission in alopecia universalis while receiving physiologic glucocorticoids and mineralocorticoid, rituximab, and chronic therapy with methotrexate. In APS1, immunosuppressive agents have been used primarily in patients with life-threatening features such as autoimmune hepatitis, pulmonary interstitial disease, or severe malabsorption. As APS1 itself is life-threatening, early consideration of immunosuppressive agents may be warranted. Early testing for adrenal insufficiency may justify early use of glucocorticoids and mineralocorticoids.

## Data Availability

The data used to support the findings of this study are available from the corresponding author upon request.
